# Study of Microbiota Associated to Early Tumors Can Shed Light on Colon Carcinogenesis

**DOI:** 10.3390/ijms252413308

**Published:** 2024-12-11

**Authors:** Anna Aspesi, Marta La Vecchia, Gloria Sala, Emilia Ghelardi, Irma Dianzani

**Affiliations:** 1 Department of Health Sciences, Università Del Piemonte Orientale, 28100 Novara, Italy; anna.aspesi@med.uniupo.it (A.A.); marta.lavecchia@uniupo.it (M.L.V.); gloria.sala@uniupo.it (G.S.); 2 Department of Translational Research and New Technologies in Medicine and Surgery, University of Pisa, 56123 Pisa, Italy; emilia.ghelardi@unipi.it

**Keywords:** gut microbiota, colorectal cancer, colon polyp, lumen-associated microbiota, mucosa-associated microbiota, intestinal pathogens

## Abstract

An increasingly important role for gut microbiota in the initiation and progression of colorectal cancer (CRC) has been described. Even in the early stages of transformation, i.e., colorectal adenomas, changes in gut microbiota composition have been observed, and several bacterial species, such as pks^+^ *Escherichia coli* and enterotoxigenic *Bacteroides fragilis*, have been proposed to drive colon tumorigenesis. In recent years, several strategies have been developed to study mucosa-associated microbiota (MAM), which is more closely associated with CRC development than lumen-associated microbiota (LAM) derived from fecal samples. This review summarizes the state of the art about the oncogenic actions of gut bacteria and compares the different sampling strategies to collect intestinal microbiota (feces, biopsies, swabs, brushes, and washing aspirates). In particular, this article recapitulates the current knowledge on MAM in colorectal adenomas and serrated polyps, since studying the intestinal microbiota associated with early-stage tumors can elucidate the molecular mechanisms underpinning CRC carcinogenesis.

## 1. Introduction

The human gastrointestinal tract harbors trillions of microorganisms, collectively referred to with the term microbiota, that play key roles in the digestion of complex polysaccharides, the elimination of toxic substances and pathogens, and the modulation of host metabolism and immunity [1]. Dysbiosis, i.e., the disruptions in the taxonomic and metabolic balance of the intestinal microbial community, is associated with a number of pathologies, including obesity [2,3], type 2 diabetes mellitus [4], inflammatory bowel disease [5,6], cardiovascular disease [7,8] and cancer [9]. Accumulating evidence indicates that the gut microbiota is especially involved in the initiation and progression of colorectal cancer (CRC). CRC is a leading cause of death worldwide, and its incidence is predicted to increase to 3.2 million new cases and 1.6 million deaths by 2040 [10]. The etiological factors of CRC are complex and heterogeneous and involve both non-modifiable and modifiable risk factors. The non-modifiable factors include age, male sex [11], and genetic predisposition, which can be due to high-penetrance germline mutations that are found in a small proportion (3–5%) of CRC patients [12] or to the combined effect of low-penetrance alleles [13]. The modifiable risk factors for CRC are mostly associated with dysbiosis and inflammation and include high consumption of animal fat, red and/or processed meat, low intake of fiber-rich foods, and a sedentary lifestyle [14].

In most cases (70–90%), CRC develops from epithelial cells through the acquisition of genetic and epigenetic alterations that lead first to hyperproliferation and then to tumor initiation, via the so-called adenoma–carcinoma sequence, a series of well-defined molecular and histopathological changes [15]. The early lesions, benign adenomatous polyps, typically present mutation/inactivation of the tumor suppressor adenomatous polyposis coli (APC), which results in stabilization of β-catenin and activation of Wnt/β-catenin signaling [16]. The accumulation of mutations in other genes, such as *KRAS* and *TP53*, causes the outgrowth of more malignant cells and the progression of benign polyps to tubular adenomas (TAs) with increasing grade of dysplasia and eventually to invasive adenocarcinomas [17]. These tumors exhibit chromosomal instability (CIN), with aneuploidy and large chromosomal aberrations. Alternatively to the conventional adenoma–carcinoma pathway, 10–30% of all CRCs evolve along the serrated pathway. The molecular alterations that characterize these tumors are the CpG island methylator phenotypes (CIMPs), due to genome-wide promoter hypermethylation and silencing of a wide range of tumor suppressor genes, *BRAF* activating mutations, but rarely *APC* mutations [18,19]. The precursor lesions of the serrated pathway are histologically classified into benign hyperplastic polyps (HPs), sessile serrated adenomas/polyps (SSA/Ps), and traditional serrated adenomas (TSAs) [20,21].

Many studies have demonstrated that the gut microbiota is significantly altered in CRC patients compared to healthy subjects, and diverse bacteria whose abundance correlates with tumor presence have been proposed as diagnostic markers [22,23,24]. Moreover, functional studies on animal models have corroborated the causal association between microbiota alterations and CRC.

This review discusses the current knowledge of how gut microbiota can affect the development and progression of early colon neoplasms.

## 2. Intestinal Bacteria Can Act as Oncogenic Factors

**Figure 1:**
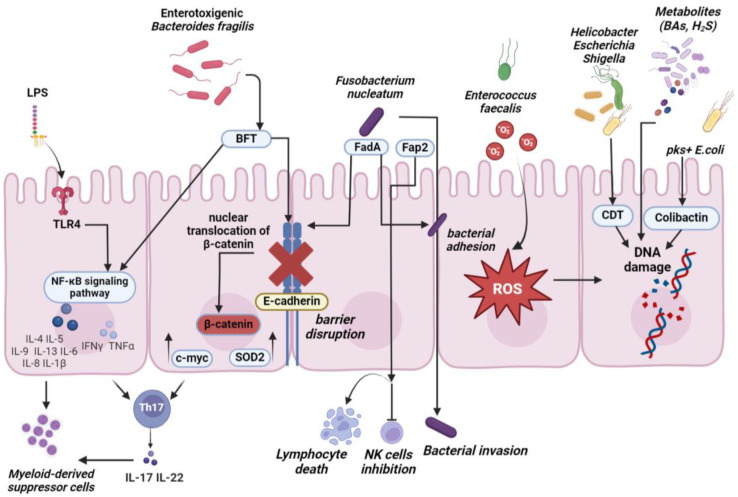
The diverse mechanisms used by pathobionts to induce damage. The information included in this figure refers to citations [28,29,30,31,32,33,34,35,36,37,38,39,40,41,42,43,44,45,46,47,48,53,54,55,56,57,64,65,66,67] and was created with BioRender.com app.biorender.com (accessed on 15 October 2024).

Recent studies have elucidated the main mechanisms by which intestinal bacteria can influence the initiation and progression of CRC, which are by the production of bacterial toxins, the release of metabolites, and the modulation of inflammation and immune responses [25] (Figure 1).

Several intestinal bacteria can secrete genotoxins and virulence factors that lead to DNA mutagenesis or functional damage in the host cells [26,27]. Cytolethal distending toxin (CDT), which is produced by a large number of gram-negative bacteria, including *Helicobacter*, *Escherichia* and *Shigella* species, is composed of three subunits, i.e., CdtA, CdtC, and the catalytically active CdtB [28,29]. CDT causes single and double-strand DNA breaks in eukaryotic cells, which are associated with cell cycle arrest, apoptosis, or mutagenesis if the DNA lesion is unrepaired or misrepaired [30,31].

Colibactin is a genotoxin produced by *Escherichia coli* strains harboring the polyketide synthase genomic island (pks^+^ *E. coli*) [32]. It has been demonstrated that colibactin can cause DNA double-strand breaking, chromosome aberrations, DNA alkylation and production of DNA adducts, and prolonged cell cycle arrest [33,34].

Enterotoxigenic *Bacteroides fragilis* (ETBF) can secrete the virulence determinant *B. fragilis* toxin (BFT), a metalloprotease able to induce the cleavage of the extracellular domain of E-cadherin, which is a key component of adherent junctions. E-cadherin cleavage results in the translocation of β-catenin into the nucleus and in the expression of the proto-oncogene c-myc [35]. BFT activates the Wnt/β-catenin and nuclear factor-κB (NF-κB) signaling pathways in colonic epithelial cells, leading to increased cell proliferation, barrier disruption, and production of inflammatory mediators [35,36]. ETBF infection also promotes intestinal inflammation and colorectal carcinogenesis by downregulation of miR-149-3p expression and subsequent superoxide dismutase 2 (SOD2) overexpression [37]. The *bft* gene was detected with higher frequency in mucosal and stool samples of CRC cases compared to controls [38,39]; *bft* positivity was significantly increased in advanced- vs. early-stage CRC patients.

The gram-positive gut commensal *Enterococcus faecalis* can generate extracellular superoxide, which promotes DNA damage and chromosomal instability in colonic epithelial cells. Superoxide upregulates cyclooxygenase-2 (COX2) expression in macrophages leading to the production of 4-hydroxy-2-nonenal, which favors malignant transformation in IL-10 knock-out mice [40,41,42].

Many studies strongly support the involvement of *Fusobacterium*, especially *Fusobacterium nucleatum*, in CRC [24,43,44]. Inoculation of *F. nucleatum* into Apc^Min/+^ mice results in an NF-κB pro-inflammatory signature and in accelerated onset of small intestinal and colonic tumors in the absence of colitis or macroscopical inflammation [45]. *F. nucleatum* expresses on its surface the FadA adhesion protein, which binds to E-cadherin and activates β-catenin signaling further leading to cell proliferation [46]. Another virulence factor expressed by *F. nucleatum* is the Fap2 protein, which can cause human lymphocyte death [47] and inhibit the activities of NK cells and T cells through interaction with their TIGIT receptor [48].

Gut bacteria can also affect colon cell fate and influence host homeostasis by producing a plethora of molecules, such as secondary bile acids (BAs) and short-chain fatty acids (SCFAs). In the liver, the primary BAs chenodeoxycholic acid and cholic acid are produced from cholesterol, conjugated to taurine or glycine to form bile salts, and excreted into the duodenum to aid fat digestion [49]. In the distal small intestine and in the colon, gut bacteria can deconjugate BAs and convert them into secondary BAs, namely lithocholic and deoxycholic acid, which can induce inflammation through activation of the transcription factor NF-κB in colonic epithelial cells and act as tumor-promoting metabolites [50]. Clinical studies have shown that high-fat diets increase secondary BAs production [51,52] and that high concentrations of BAs are correlated to increased risk of CRC [53,54,55]. Administration of deoxycholic acid induces intestinal inflammation and disrupts the mucosal barrier in *Apc*^min/+^ mice [56] and facilitates tumorigenesis in rats treated with azoxymethane (AOM), a colorectal carcinogen [57]. Conversely, increasing evidence indicates that SCFAs such as butyrate have antineoplastic properties since they maintain mucosal integrity, inhibit colonic inflammation, and reduce CRC risk [58,59]. Butyrate is the preferred energy source for normal colonocytes, whereas cancerous colonocytes rely on glucose to produce energy, and accumulate butyrate, which functions as a histone deacetylase (HDAC) inhibitor, leading to suppression of cell proliferation and tumor development [60,61].

Finally, pathogenic bacteria can induce host cell damage by regulating the functions of immune cells. Under normal physiological conditions, gut bacteria are of fundamental importance for the maturation of gut-associated lymphoid tissue (GALT) and for the induction of tolerance to commensal microbiota antigens [62]. Symbiotic intestinal microbes stimulate the secretion of interleukin-1 beta (IL-1β) by macrophages and thus the expansion of regulatory T cells (Tregs), which cooperate to immune homeostasis and maintenance of intestinal mucosal integrity [1]. A healthy microbiota promotes the anti-tumoral functions of cytotoxic CD8+ T cells, which can recognize tumor antigens presented on the surface of transformed cells and eliminate tumor cells via release of cytotoxic granules and secretion of pro-inflammatory cytokines [63]. Conversely, dysbiosis can stimulate excessive production of pro-inflammatory cytokines, promote epithelial cell proliferation, and limit the action of antitumorigenic immune cells [25]. Specific bacteria can indeed suppress the beneficial actions of immune cells, leading to cancer development. A paradigmatic example, in addition to the already mentioned *F. nucleatum*, is offered by ETBF, which induces chronic colitis and colon tumorigenesis in murine models via activation of T helper type 17 (Th17) cells, release of IL-17, and differentiation of myeloid cells into immunosuppressive myeloid-derived suppressor cells [64,65]. Moreover, lipopolysaccharide (LPS), a component of the outer membrane of gram-negative bacteria binds to the toll-like receptor 4 (TLR4) on the surface of colonic epithelial cells and activates the NF-κB signaling pathway, resulting in the production of numerous chemokines and pro-inflammatory cytokines [66,67].

To explain how the oncogenic potential of certain bacteria can result in CRC development, Sears and Pardoll proposed the “Alpha-bug” hypothesis, which states that specific microbes with unique virulence traits not only can have genotoxic effects on colonocytes but can also modulate the colonic bacterial community to generate a prooncogenic environment that favors neoplastic transformation [68]. Tjalsma et al. extended this hypothesis by proposing the “driver-passenger” model for colon carcinogenesis, where driver bacteria with procarcinogenic features that may contribute to CRC initiation are progressively outcompeted and possibly replaced by bacteria more suited to grow in the tumor microenvironment [69]. This model implies that tumor progression is accompanied by remodeling of gut microbiota and explains the observation that microbiota composition is different in patients carrying early lesions, i.e., adenomas, compared to patients with CRC [70,71]. By focusing on the bacterial communities associated to colorectal adenomas, it should be possible to identify the microbes that drive tumorigenesis rather than the bacteria that display a growth advantage in the CRC environment, which is characterized by profound metabolic alterations, including enhanced glycolysis, lower pH, and elevated amino acids concentration [72].

## 3. The Dilemma of Sampling Gut Microbiota

**Figure 2:**
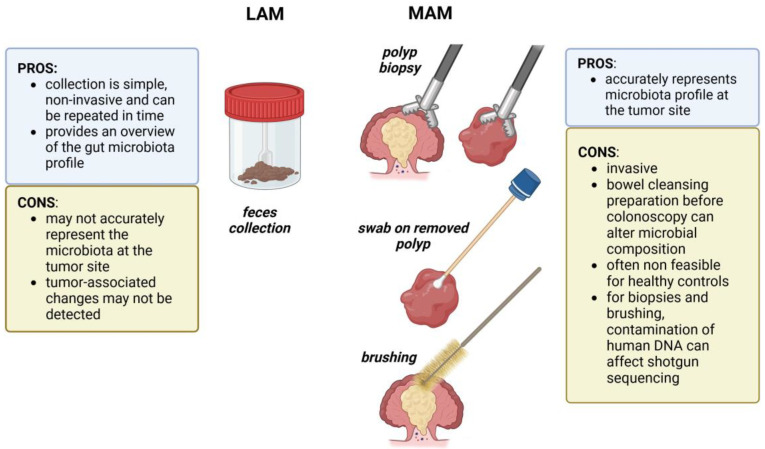
The most commonly employed methods to collect samples for gut microbiota studies. The information included in this figure refers to citations [73,74,75,76,81,82,83,84,85,86] and was created with app.biorender.com (accessed on 15 October 2024).

In recent years, advances in next-generation sequencing methodologies and bioinformatics tools have been instrumental in providing a better understanding of microbiota composition, but particular attention should be devoted to the choice of specimens for sequencing analyses (Figure 2). Stools are frequently used for intestinal microbiota studies since their collection is simple and noninvasive. However, fecal samples do not provide information on the distribution of bacteria in different districts of the intestinal tract, nor do they reflect the microbial communities in close contact with the epithelium. The lumen-associated microbiota (LAM), represented by feces, and the mucosal-associated microbiota (MAM) that can be studied by collecting mucosal biopsies during colonoscopy, are two distinct ecosystems that differ significantly from each other in microbial diversity and composition [73,74,75,76]. Many independent studies showed that MAM derived from biopsies was significantly less diverse than LAM [73,74,77,78]. For instance, in a study on individuals undergoing routine screening colonoscopies, either healthy or with hyperplastic polyps or tubular adenomas, it has been observed that the MAM had reduced species richness (evaluated by Chao1 index) and diversity (by Shannon index) compared to the LAM [79]. The predominant phylum was *Proteobacteria* in MAM and *Firmicutes* in LAM [79]. It has been proposed that an increased level of oxygen-tolerant organisms of the *Proteobacteria* phylum may be present in the mucosa because of the different oxygen content which is higher in the mucosal interface and lower in the lumen [80].

The microbes adherent to the surface of the intestinal mucosal cells are considered the most important for the fortification of host immune defenses and other beneficial functions but also for procarcinogenic processes, such as inflammation [81,82]. Mucosal biopsies obtained during colonoscopy have been used to study MAM in different anatomical sites and in tumors. This approach presents the disadvantages of being invasive for the patients and being influenced by the unavoidable alterations due to bowel preparation [78,83,84]. An alternative strategy to biopsies is the collection of MAM by swabs, brushes, or washing aspirates. Avelar-Barragan and coll. tested multiple sampling methods to obtain MAM during colonoscopies from subjects with TAs, HPPs, SSPs, or healthy controls. In particular, they directly brushed the surface of polyps and of normal tissue on the opposite colon wall and collected the colonoscopy washing fluid sprayed on normal tissue near the polyps. Both techniques gave similar microbiome profiles, but samples collected by brushing resulted in higher proportions of human-derived reads during shotgun sequencing and in a higher risk of damaging the intestinal epithelium. MAM obtained by mucosal aspirates had significantly decreased species richness and Shannon diversity than LAM analyzed in fecal samples [85]. In 2019, we developed a novel approach to collect both MAM and mucosa-associated metabolites from the tumor surface [77,86]. This method consists in gently brushing swabs on the surface of colorectal polyps after their removal during colonoscopies, ensuring the collection of bacteria and metabolites present on the tumor surface and not on the normal mucosa nearby. Moreover, this approach preserves the integrity of the polyps and does not interfere with histopathological analyses. MAM was subsequently analyzed by 16S rDNA sequencing and compared to LAM from the same patients, obtaining comparable number of taxa from MAM and LAM samples (165 and 202 taxa, respectively) but differences in diversity and composition [77]. This method is suitable also for shotgun sequencing and is expected to avoid human reads contamination. Shotgun sequencing has the advantage, compared to 16S rDNA sequencing, to have higher resolution (even down to strain identification) and to infer bacterial functions. However, since with shotgun metagenomics all the DNA (and not only the 16S amplicons) is sequenced, host DNA is a considerable contaminant, especially for biopsies. For this reason, host DNA depletion methods are developing [87]. Other innovative biotechnological strategies allow the identification of promising biomarkers (e.g., metabolites, miRNAs) from various biological fluids and tissues, including feces [77,88,89,90].

Overall, MAM collection (by biopsies, swabs, brushes, or washing aspirates) is invasive and not always feasible for healthy individuals, and its composition is influenced by colonoscopy preparation. Swabs, brushes, and washing aspirates, differently from biopsies, have the advantage of preserving polyp integrity; biopsies and brushes are the MAM collection methods that are more affected by host DNA contamination. On the other hand, although fecal samples are a powerful resource for biomarker discovery and application in CRC screening since stool collection is easy and not invasive, it seems reasonable to consider MAM more representative of the complex ecosystem that drives transformation of colonic epithelial cells. Indeed, fecal samples provide an overview of the gut bacterial environment but do not represent tumor-site microbiota as accurately as MAM.

## 4. Mucosa-Associated Bacteria in Patients with Colon Adenomas

Some studies focused on the bacterial composition of rectal mucosa in patients carrying colon adenomas vs. adenoma-free controls [91,92,93,94] under the assumption that the microbiota is relatively stable along the digestive tract [95,96] and that the rectal mucosa might be considered a proxy of the mucosa at the adenoma site. Microbial richness of the rectal mucosa was increased in subjects with adenomas compared to controls [91,94]. A significantly higher abundance of *Proteobacteria* and lower abundance of *Bacteroidetes* was observed in cases with adenoma compared to controls [94]. The relative abundance of potential pathogens such as *Pseudomonas*, *Helicobacter*, *Acinetobacter*, and other genera belonging to the phylum *Proteobacteria* was significantly increased in cases [91], as well as *Fusobacterium* and *Bifidobacterium* spp. [92,93]. Moreover, Shen et al. observed that some less abundant genera, i.e., *Oscillospira* spp., *Clostridium* spp., *Phascolarctobacterium* spp., *Finegoldia* spp., *Eubacterium* spp., and *Akkermansia* spp. were present only in cases but not in controls [94].

Several other studies compared the microbiota of adenoma biopsies to the surrounding healthy tissue. The *Fusobacterium* spp. level was measured by qPCR in adenoma biopsies and adjacent normal tissue from the same patients. *Fusobacterium* was found present in 48% of adenomas and was significantly enriched in adenomas compared to the adjacent tissue [45]. This suggests that *Fusobacterium* begins to accumulate at early stages of colon tumorigenesis.

Lu et al. compared the bacterial composition of biopsies from adenoma and normal adjacent tissue collected from 31 patients with adenoma and found no significant differences at the phylum level [82], a result confirmed by a subsequent report [97]. Comparison of these samples to the colon biopsies collected from 20 healthy volunteers showed a remarkably different microbiota [82]. In particular, a conspicuous reduction in Firmicutes with concomitant expansion of *Proteobacteria* was observed in patients with adenomas, and the *Firmicutes*/*Bacteroidetes* ratio, which is considered a marker of eubiosis in the gastrointestinal tract, was decreased [82]. Biopsies of premalignant polyps also had a higher abundance of *Bifidobacterium*, *Faecalibacterium*, *Bacteroides*, and *Romboutsia* than the healthy colon mucosa isolated from the same patients and reduced levels of *Helicobacter* and *Klebsiella* [97]. Since *Faecalibacterium*, *Bacteroides*, and *Romboutsia* are also depleted in CRC mucosa, these taxa may represent microbial biomarkers associated with the presence of either early or advanced tumor lesions [97].

Mira-Pascual et al. found that TA samples had increased diversity compared to adjacent normal tissue, and the diversity was even higher in CRC samples [98].

A paper by Nakatsu et al. described the microbial communities in 47 cases with colorectal adenomas, 52 cases with invasive adenocarcinomas, and 61 controls without colorectal tumors [99]. Biopsies were obtained from tumors and tumor-adjacent mucosa and analyzed by 16S ribosomal RNA gene sequencing to determine associations of distinct taxonomic configurations with disease status. No statistical difference in microbial diversity was found between tumors and tumor-adjacent mucosa, but carcinoma samples had a significant increase in diversity when compared to adenomas. Adenomatous lesions showed signs of dysbiosis and the enrichments of *E. coli* [99]. Data obtained in this study [99] were also used by Xu et al., who compared microbiota of mucosa biopsies from CRC cases, adenoma cases, and healthy controls, and found a significant enrichment of *Fusobacteria* in patients with CRC compared to patients with adenomas and control subjects. No significant difference at the phylum and genus levels was found between the normal and adenoma groups. The genus *Escherichia* was more abundant in adenoma patients than in CRC patients and healthy controls [100]. The authors hypothesize that *E. coli* might colonize the colon mucosa and act as a driver of tumorigenesis then be outcompeted by passenger bacteria that acquire a growth advantage in the tumor microenvironment. The authors propose *E. coli* as a candidate adenoma-associated biomarker [100].

The levels of three bacteria that have been implicated in the development of CRC, i.e., *F. nucleatum*, *B. fragilis*, and *Streptococcus gallolyticus* [24,35,36,101,102,103,104,105,106], were quantified by qPCR in biopsies from 99 patients with CRC (tumor and paired normal tissue), 96 patients with adenomas, and 104 patients with diverticula [107]. *S. gallolyticus* was detected in none of the samples. *F. nucleatum* and *B. fragilis* were significantly reduced in adenoma tissues compared to diverticula and to CRC (both tumors and paired normal tissues). The genus *Acinetobacter* was highly abundant in both diverticula and adenomas but absent in samples derived from CRC patients, while the genus *Prevotella* was associated to CRC [107]. It is well known that some strains of *Prevotella* can promote chronic inflammation by driving Th17-mediated immune responses [108,109].

Comparison of biopsies from 15 patients with adenomatous polyps and 46 CRC patients showed a reduction in the families of *Campylobacteraceae*, *Carnobacteriacerae*, *Gemellaceae*, *Leptotrichiaceae*, and *Streptococcaceae*, and an increase in *Pseudomonadaceae* and *Yersiniaceae* in adenoma vs. CRC [71]. At the genus level, a reduced level of *Fusobacterium* and *Gemella* and an increased level of *Pseudomonas* and *Serratia* were found in adenomas compared to CRC [71].

Geng et al. relied on the driver-passenger model [69] to interpret the results obtained on 10 normal, 10 adenomas, and eight CRC biopsy samples. Taxa, whose relative abundances in adenoma tissues were significantly higher than in normal and CRC tissues, such as *Enterobacteriaceae*, *Pseudomonadaceae*, *Neissenaceae*, and *Enterobacter*, were classified as potential drivers. The family *Streptococcaceae* and the genus *Streptococcus* were considered possible pro-inflammatory passengers [110].

A study on a small cohort of Norwegian patients evaluated by quantitative PCR (qPCR) the levels of *F. nucleatum* and four *E. coli* toxin genes in biopsies from 21 CRC patients, 11 adenoma patients, and 11 healthy controls [111]. The levels of *E. coli* toxin genes in stool and biopsy samples were not significantly different among groups. *F. nucleatum* was more frequently detected in biopsies from CRC patients, and significantly higher levels of *F. nucleatum* and *Fusobacterium* spp. were identified in stool samples from CRC patients compared with adenoma patients and healthy controls [111]. Similarly, a study on biopsies from nine CRC patients with synchronous adenomas, 16 colorectal adenoma (CRA) patients, and 10 healthy subjects, showed that *F. nucleatum* was significantly enriched in tumor, adenoma, and normal adjacent tissues from CRC patients compared to healthy controls but not in adenoma and normal adjacent tissues from CRA patients [112]. Table 1 summarizes the mentioned articles.

**Table 1:** Summary of the literature data about mucosa-associated bacteria in patients with colon adenomas.

Reference	Samples	Comparison	Results
[91]	Biopsies from rectal mucosa	33 subjects with adenomas vs. 38 adenoma-free controls	*Pseudomonas*, *Helicobacter*, *Acinetobacter*, and other genera belonging to the phylum *Proteobacteria* increased in cases.
[94]	Biopsies from rectal mucosa	21 subjects with adenomas vs. 23 non-adenoma controls	Higher richness and higher abundance of *Proteobacteria* and lower abundance of *Bacteroidetes* in cases. *Oscillospira* spp., *Clostridium* spp., *Phascolarctobacterium* spp, *Finegoldia* spp., *Eubacterium* spp., and *Akkermansia* spp. present only in cases.
[45]	Adenoma/colon biopsies	Adenoma biopsies vs. surrounding healthy tissues of 29 patients	*Fusobacterium* enriched in adenomas.
[82]	Adenoma/colon biopsies	Adenoma biopsies vs. surrounding healthy tissues of 31 patients	No significant differences at the phylum level.
		Adenoma biopsies of 31 patients vs. colon biopsies collected from 20 healthy volunteers	Reduction in *Firmicutes* and expansion of *Proteobacteria* in patients with adenomas.
[97]	Adenoma/colon biopsies	Premalignant polyp biopsies vs. surrounding healthy tissues of 12 patients	Higher abundance of *Bifidobacterium*, *Faecalibacterium*, *Bacteroides*, and *Romboutsia* and reduced levels of *Helicobacter* and *Klebsiella* in premalignant polyps.
[98]	Adenoma/colon biopsies	Adenomas vs. adjacent normal tissues of 7 subjects with CRC and 11 with tubular adenomas	Increased diversity in adenomas and CRC compared to normal tissue.
[99]	Adenoma/colon biopsies	47 cases with colorectal adenomas, 52 cases with invasive adenocarcinomas, and 61 controls without colorectal tumors	Increased diversity in carcinomas compared to adenomas. Enrichment of *E. coli* in adenomas.
[100]	Data from [99]	Data from [99]	Enrichment of *Fusobacteria* in patients with CRC compared to patients with adenomas and control subjects. No differences between normal and adenoma groups. *Escherichia* more abundant in adenoma patients than in CRC patients and healthy controls.
[107]	Adenoma/colon biopsies	99 patients with CRC (tumor and paired normal tissues), 96 patients with adenomas, and 104 patients with diverticula	*F. nucleatum* and *B. fragilis* reduced in adenoma tissues compared to diverticula and to CRC. *Acinetobacter* highly abundant in both diverticula and adenomas. *Prevotella* associated to CRC.
[71]	Adenoma/colon biopsies	15 patients with adenomatous polyps vs. 46 CRC patients	Reduction in the families of *Campylobacteraceae*, *Carnobacteriacerae*, *Gemellaceae*, *Leptotrichiaceae*, and *Streptococcaceae* and increase in *Pseudomonadaceae* and *Yersiniaceae* in adenoma vs. CRC. Reduced level of *Fusobacterium* and *Gemella* and increased level of *Pseudomonas* and *Serratia* in adenomas compared to CRC.
[110]	Adenoma/colon biopsies	10 normal, 10 adenoma, and 8 CRC	Higher *Enterobacteriaceae, Pseudomonadaceae, Neissenaceae*, and *Enterobacter* in adenoma tissues and reduced *Streptococcus*.
[111]	Adenoma/colon biopsies	21 CRC patients, 11 adenoma patients, and 11 healthy controls	*F. nucleatum* more frequently detected in biopsies from CRC patients.
[112]	Adenoma/colon biopsies	9 CRC patients with synchronous adenomas, 16 colorectal adenoma (CRA) patients, and 10 healthy subjects	*F. nucleatum* enriched in tumor, adenoma, and normal adjacent tissues from CRC patients compared to healthy controls, but not in adenoma and normal adjacent tissues from CRA patients.

## 5. Mucosa-Associated Bacteria Involved in the Serrated Pathway

Only a few studies have compared the microbiomes profiles of premalignant colorectal lesions developed through the traditional adenoma–carcinoma sequence and the serrated pathway. Since the genetic and epigenetic changes underlying CRC carcinogenesis are different for these two pathways, it is possible that distinct microbes play a specific role for each pathway. Burns and coll. found that tumors with *APC* mutations, a feature typical of the adenoma–carcinoma sequence but not of the serrated pathway, correlate with an increase in abundance of the genus *Finegoldia*, which is an opportunistic pathogen highly prevalent in skin wounds [113,114].

A paper reported no significant difference in MAM among healthy controls, patients with conventional adenoma, SSA, and CRC, but two important limits of this work were the small number of subjects (24 in total) and the fact that the MAM was examined from biopsy samples of normal rectal mucosa, not of tumors [115].

Park et al. analyzed by 16S rDNA sequencing the MAM from TA, SSA/P, and CRC biopsy samples and noticed that *Fusobacteria* was identified in 37.5% of TAs, 50% of SSA/Ps, and 100% of CRCs. The relative abundance of *Fusobacteria* was similar between the TA and SSA/P groups but was significantly higher in the CRC group [116]. Noteworthy, *Fusobacteria* was also identified in biopsies of normal tissue adjacent to the neoplastic lesions. These results suggest that *Fusobacteria* may contribute to tumorigenesis via both the adenoma–carcinoma sequence and the serrated pathway [116] and are in good agreement with previous work by Ito et al., who evaluated by qPCR the level of *F. nucleatum* in 138 HPs, 129 SSAs, 102 TSAs, 131 non-serrated adenomas, and 544 CRCs. *F. nucleatum* was detected in 56% of CRCs and in 24–35% of premalignant colorectal lesions with no significant association with histopathology. Moreover, *F. nucleatum* positivity was significantly associated with CIMP-high status and larger size of premalignant lesions. The presence of *F. nucleatum* in SSAs gradually increased from the sigmoid colon to the ascending colon and cecum [117].

Avelar-Barragan et al. compared the MAM of polyp-free controls vs. patients with TAs vs. patients with serrated polyps (HPP, TSA, or SSP) by metagenome analysis of mucosal aspirates, i.e., the colonoscopy fluid washed on the mucosa near the polyp. No significant difference in Shannon diversity or richness was observed among groups [85]. Patients with TA showed an enrichment of *Lachnospiraceae*, such as *Ruminococcus gnavus*, which has been previously associated with inflammatory bowel disease [118,119], and *Clostridium scindens*, which can transform primary BAs into secondary BAs [120]. The bacterium *Eggerthella lenta* was significantly less abundant in serrated polyps compared to aspirates from healthy controls. This bacterium metabolizes inert plant lignans into bioactive enterolignans with antiproliferative and anti-inflammatory effects [121,122]. This finding is coherent with the hypothesis that a low-fiber diet can favor aberrant epigenetic alterations in colonic epithelial cells and induce development of serrated polyps [85].

## 6. Discussion: Future Directions and Perspectives

Dysbiosis can contribute to carcinogenesis by promoting cell proliferation, inflammation, and DNA damage [123].

Many studies investigating the composition of microbial communities in CRC have relied on fecal samples, mainly because they can be exploited for cancer screening and early detection. Although convenient and noninvasive, fecal samples reflect the luminal microbial community and do not fully capture the microbiota adherent to the mucosal layer, which is more closely associated with cancer development. The microbiota directly in contact with the tumor surface can be analyzed from biopsies or mucosal swab/brushes, but these methods require invasive procedures. Moreover, biopsies and mucosal brushes contain a large proportion of human-derived reads when analyzed by shotgun sequencing, and this can affect data quality [85,87].

There is an urgent need for prospective longitudinal studies that address the role of dysbiosis in the etiopathogenesis of CRC by a thorough characterization of the risk factors (e.g., unhealthy diet, sedentary life, and comorbidities) [124] in large cohorts of subjects. A clear understanding of the temporal relationship between microbiota alterations and carcinogenesis might allow the identification of a gut bacterial signature that defines individuals with high risk of CRC. This would be of crucial importance for the implementation of preventative and therapeutic measures based on microbiota manipulation.

Treatments with immune checkpoint inhibitors (ICIs) have been proven effective for CRC patients with DNA mismatch repair deficiency or high microsatellite instability (MSI-H) but mostly unsuccessful for patients without these characteristics [125,126,127]. It has been demonstrated that microbiota modulation can improve the efficacy of immunotherapy for patients with CRC and other tumors [128,129]. Administration of specific bacteria, such as *Bifidobacterium*, *Akkermansia muciniphila*, and *Faecalibacterium prausnitzii*, positively influences immune responses to ICIs in animal models [128,130,131].

Possible modulation strategies in CRC patients include dietary interventions, probiotics, prebiotics, and fecal microbiota transplantation (FMT). FMT has emerged in recent years as a promising option [132], especially after the outstanding results obtained to treat *Clostridioides difficile* infections (CDI). Clinical studies demonstrated that FMT is highly effective in treating recurrent CDI, with success rates around 80–90% for patients that do not respond to standard antibiotic therapies [133,134,135]. More efforts are needed to clarify the potential benefits of FMT in the prevention and treatment of colorectal tumors [136].

## 7. Conclusions

An accurate representation of the microbial communities at the tumor site, with a focus on early-stage tumors, is imperative to determine how the complex interactions between microbes and host cells contribute to the etiopathogenesis of CRC. Particular attention should be directed to the study of specific bacterial species that can act as oncomicrobes and whose effects can be elucidated only by functional experiments.
